# Isolation of tissue-resident endothelial stem cells and their use in regenerative medicine

**DOI:** 10.1186/s41232-019-0098-9

**Published:** 2019-05-08

**Authors:** Tomohiro Iba, Hisamichi Naito, Shota Shimizu, Fitriana Nur Rahmawati, Taku Wakabayashi, Nobuyuki Takakura

**Affiliations:** 1 Department of Signal Transduction, Research Institute for Microbial Diseases, Osaka University, 3-1 Yamada-oka, Suita, Osaka 565-0871 Japan; 2 Division of Signal Transduction, Immunology Frontier Reserch Center, Osaka University, Suita, Japan

**Keywords:** Side population, Endothelial cell, Adipose tissue, Angiogenesis, Stem cell

## Abstract

**Background:**

During sprouting angiogenesis, stalk cells, localized behind tip cells, generate endothelial cells (ECs) for the elongation of new vessels. We hypothesized that stalk cells may have endothelial progenitor cell properties because of their highly proliferative ability. We conducted Hoechst dye DNA staining in ECs of preexisting blood vessels from hind limb muscle and found that endothelial-side population (E-SP) cells, which efflux Hoechst rapidly with abundant ABC transporters, show highly producing ability of ECs. We previously showed the existence of E-SP cells in hind limb muscle, retina, and liver, but not in other tissues such as adipose tissue, skin, and placenta.

**Methods:**

We investigated the existence of E-SP cells and analyzed their proliferative ability among CD31^+^CD45^−^ ECs from adipose tissue, skin, and placenta of adult mice. We also analyzed the neovascular formation of E-SP cells from adipose tissue in vivo.

**Results:**

We detected E-SP cells in all tissues examined. However, by in vitro colony formation analysis on OP9 cells, we found that E-SP cells from adipose tissue and skin, but not from placenta, have highly proliferative ability. Moreover, E-SP cells from adipose tissue could contribute to the neovascular formation in hind limb ischemia model.

**Conclusion:**

The adipose tissue and skin are available sources to obtain endothelial stem cells for conducting therapeutic angiogenesis in regenerative medicine.

## Background

Blood vessel formation is essential for tissue regeneration and for tissue/organ homeostasis, not only for supplying oxygen and nutrient but also for tissue-specific morphogenesis in regenerated tissues and organs [1].

Blood vessel formation consists of two processes: vasculogenesis, de novo blood vessel formation usually observed in embryos, and angiogenesis, remodeling of preexisting blood vessels especially neovascular branching by sprouting [2, 3]. The molecular mechanisms of how endothelial cells (ECs) develop, proliferate, migrate, and adhere has been clarified [4, 5], and key molecules such as the vascular endothelial growth factor (VEGF), hepatocyte growth factor (HGF), or basic fibroblast growth factor (bFGF) have been used in clinical applications for ischemic diseases [6–8].

Beside vascular regeneration therapies using molecules, trials using mesenchymal stem cells or endothelial progenitor cells (EPCs) have also been conducted [9–12]. However, the most effective therapy for ischemic diseases has not been determined, partly because there are no randomized clinical studies for comparison.

On the other hand, in case of sustainable organ regeneration, a tissue-specific stem cell population is required for cell therapies. Although tissue progenitors or terminally differentiated tissue cells may transiently recover the organ function once incorporated in the preexisting organ, sustainable tissue regeneration requires stem cells which can turn over dead cells by continuously generating tissue cells.

We reported the existence of an endothelial stem cell population in side population (SP) cells of pre-existing blood vessels, which express the high amount of ATP-binding cassette (ABC) transporter and efflux DNA dye Hoechst rapidly after uptake [13]. Endothelial (E)-SP cells generate abundant ECs, form vascular network structures in vitro, and contribute to the generation of functional blood vessels in vivo.

We also reported the existence of E-SP cells in hind limb muscle, retina, lung, and liver [13–16]; however, if available, other organs, easier to access, would be preferred to obtain endothelial stem cells. Therefore, in our study, we chose adipose tissue, skin, and placenta as candidate tissues and analyzed E-SP cells in those tissues.

## Methods

### Mice

C57BL/6 and C57BL/6-Tg (CAG-EGFP) mice (6 to 12 weeks old) were purchased from Japan SLC (Shizuoka, Japan). All experimental procedures in this study were approved by the Institutional Animal Care and Use Committee of Osaka University. Mice were handled and maintained according to the Osaka University guidelines for animal experimentation.

### Cell culture

OP9 cells (RIKEN Cell Bank, Tsukuba, Japan) were maintained in αMEM (Sigma-Aldrich Japan, Tokyo, Japan) supplemented with 20% fetal calf serum (FCS) (Sigma-Aldrich Japan), 2 mM l-glutamin (Thermo Fisher Scientific, MA, USA), and 1% penicillin/streptomycin (p/s) (Life Technologies, Tokyo, Japan). All cell lines utilized are mycoplasma free, authenticated by the supplier based on morphology and growth curve analysis, and were passaged for less than 2 months.

### Cell preparation and flow cytometry

Cells from adult mice were isolated as previously described [13]. Briefly, mice were euthanized, and organs were excised, minced, and digested with dispase II (Thermo Fisher Scientific), collagenase (Wako, Osaka, Japan), and type II collagenase (Worthington Biochemical Corp., NJ, USA) with continuous shaking at 37 °C. The digested tissue was filtered (40 μm filters) to obtain single-cell suspensions. Erythrocytes were lysed with ammonium-chloride-potassium buffer (0.15 M NH4Cl, 10 mM KHCO3, and 0.1 mM Na2-EDTA). Bone marrow cells were collected from the tibiae and femurs. Cell surface antigen staining was performed with anti-CD31 (clone MEC13.3, BD Biosciences, CA, USA) and anti-CD45 (Clone 30-F11, BD Biosciences) antibodies. Hoechst staining was performed as described previously [17]. Briefly, cell suspensions were incubated with Hoechst 33342 (5 mg/ml) (Sigma-Aldrich Japan) at 37 °C for 90 min in DMEM (Sigma-Aldrich Japan) supplemented with 2% FCS and 1 mM HEPES at a concentration of 1 × 10^6^ nucleated cells/ml in the presence or absence of verapamil (50 mM, Sigma-Aldrich Japan). Propidium iodide (PI, 2 mg/ml; Sigma-Aldrich Japan) was added before FACS analysis to exclude dead cells. Stained cells were analyzed and sorted by a SOAP FACSAria (BD Bioscience), and data were analyzed using FlowJo Software (Treestar Software, San Carlos, CA, USA).

### Primary endothelial colony forming assay

Primary ECs were isolated as described above, and 1 × 10^3^ cells/well were co-cultured with OP9 stromal cells in 24-well plates. The culture was maintained in RPMI-1640 (Sigma-Aldrich Japan), supplemented with 10% FCS (Sigma-Aldrich Japan), 10 ng/ml vascular endothelial growth factor (VEGF) (Prepro Tech, Rocky Hill, NJ) and 1% p/s. Cells were fixed for immunostaining after 10 days.

### Immunohistochemical staining

The procedure for tissue preparation and staining was previously reported [18]. For immunohistochemistry, an anti-CD31 antibody was used for staining and a biotin-conjugated polyclonal anti-rat IgG (Agilent Technologies, CA, USA) was used as the secondary antibody. Biotinylated secondary antibodies were developed using ABC kits (Vector Laboratories, CA, USA). Samples were visualized using a Canon EOS kiss X7 for the low-powered field, and a Leica DMi8 for the high-powered field. Images were processed with the Leica application suite (Leica Microsystems, Wetzlar, Germany), Adobe Photoshop CC software (Adobe Systems, CA, USA). For confocal microscopic images, sections were prepared as previously reported [19]. In brief, hind limb muscles were excised, fixed in 4% PFA/PBS, washed with PBS, embedded in OCT compound (Sakura Finetek, Tokyo, Japan), and sectioned (60 μm). The sections were stained with an anti-CD31 monoclonal antibody and anti-GFP polyclonal antibody (MBL, MA, USA). Alexa Fluor 488-conjugated anti-rat IgG and Alexa Fluor 647-conjugated anti-rabbit IgG (Invitrogen, CA, USA) were used as secondary antibodies. The sections were visualized using a Leica TCS SP5 confocal microscope and processed with the Leica Application Suite and Adobe Photoshop CC software. All images shown are representative of more than two independent experiments.

### Primary endothelial neovascularization with Matrigel

Eight-week-old mice were injected subcutaneously with 0.5 ml Matrigel (BD Bioscience) and 60 units of heparin/ml (Sigma-Aldrich Japan), 150 ng/ml VEGF, and 3000 E-SP or main population (MP) cells (E-MP cells) from the adipose tissue of EGFP mice. Fifteen days later, Matrigel plugs were removed and visualized using a Leica MZ 16 FA. Images were processed with the Leica application suite, Adobe Photoshop CC, and CLIP STUDIO PAINT (CELSYS, Tokyo, Japan).

### Hind limb ischemia model and transplantation

The hind limb ischemia model mouse was previously described [20]. Briefly, the proximal portion of the right femoral artery and vein, including the superficial and the deep branch as well as the distal portion of the saphenous artery and vein, were occluded and resected.

For the preparation of E-SP and MP cell transplantation, E-SP and MP cells were sorted from EGFP mice and diluted with DMEM (Sigma-Aldrich, Japan), supplemented with 100 ng/ml VEGF. Just after occlusion and removal of vessels, 3000 E-SP or MP cells were injected into the muscle. Two weeks later, transplanted sites were visualized. Images were taken and processed as described above.

### GFP bone marrow transplantation model

C57BL/6 mice received bone marrow (BM) transplantation as previously described [13]. In brief, BM cells were obtained by flushing the tibias and femurs of age-matched donor EGFP mice. The transplantation was performed to recipient mice lethally irradiated with 10.0 Gy, by intravenous infusion of 1 × 10^7^ donor whole BM cells. Three months after transplantation—when the BM of recipient mice was reconstituted—the mice were used for analysis.

### Statistical analysis and graphs

All data are presented as the mean ± SEM. Statistical analyses were performed using Statcel 3 (OMS, Tokorozawa, Japan). Data were compared using Student’s *t* test or one-way analysis of variance (ANOVA). *p* values < 0.05 were considered to be significant. All graphs were generated using Excel and Adobe Illustrator CC software.

## Results

### Identification of E-SP cells

**Fig. 1:**
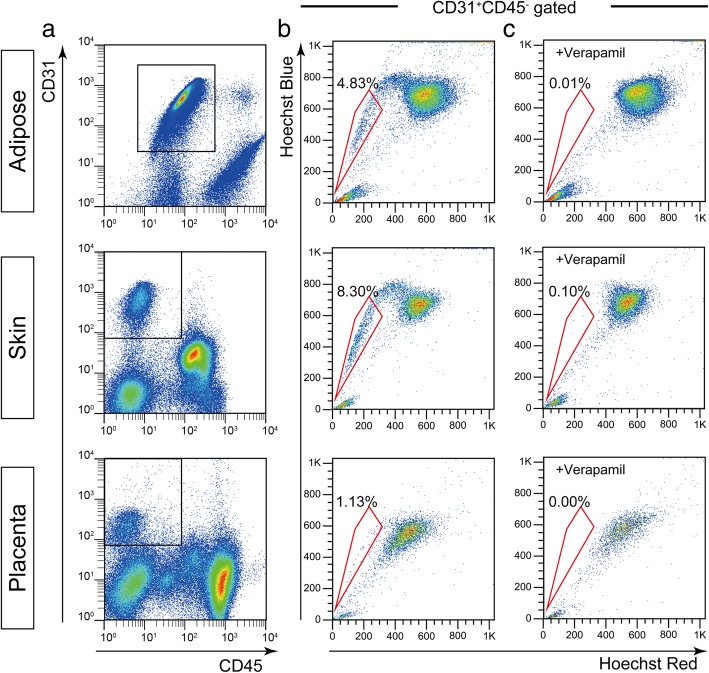
Identification of endothelial side population (E-SP) cells. **a** FACS analysis of ECs from three different murine tissues. The boxes indicate CD31^+^CD45^−^ ECs. **b** Hoechst analysis of ECs gated in **a**. The gate surrounded by red line indicates SP cells. **c** Hoechst staining of ECs in the presence of verapamil

First, we investigated E-SP cells in three different candidate tissues: the adipose tissue, skin, and placenta. CD31^+^CD45^−^ cells were recognized as ECs (Fig. 1a). E-SP cells were detected in these ECs by Hoechst analysis (Fig. 1b). We validated the SP phenotype by using verapamil, a drug efflux pump inhibitor; cells in the red gate were SP cells, because they disappeared when verapamil was used (Fig. 1c). The percentage of E-SP cells derived from the adipose tissue, skin, and placenta were 4.83 ± 0.61%, 8.30 ± 2.13%, and 1.13 ± 0.12%, respectively.

### Proliferation and colony formation of E-SP cells

**Fig. 2:**
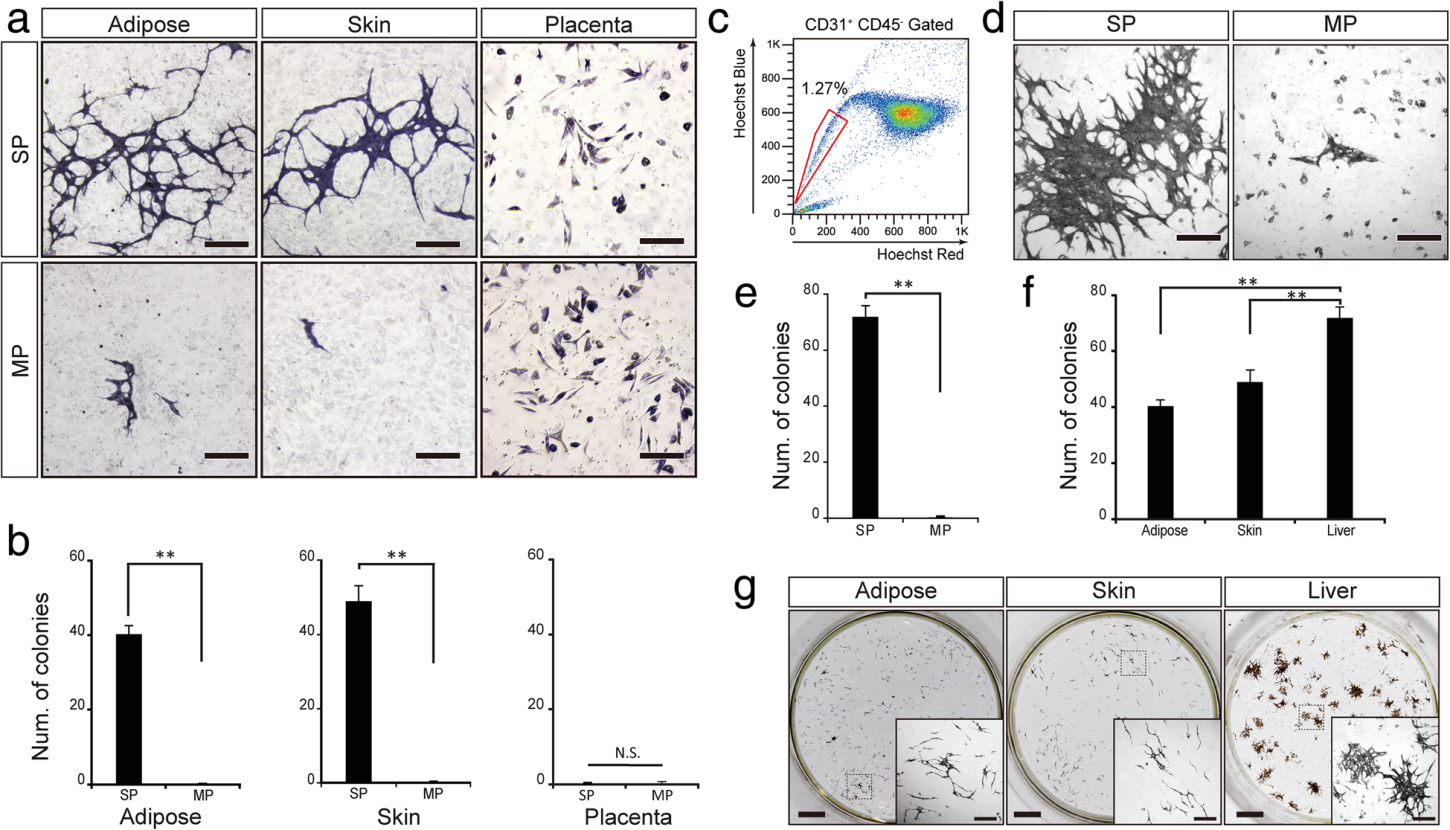
Adipose- and skin-derived E-SP cells possess colony formation ability. **a** E-SP and E-MP cells were cultured on OP9 feeder cells. These cells were stained with anti-CD31 antibody. **b** Number of colonies formed by cultured ECs. Data show mean ± SEM; **p < 0.01 (*n* > 3). **c** FACS analysis of ECs from liver. The gate surrounded by the red line indicates SP cells. **d** Liver E-SP and E-MP cells were cultured on OP9 feeder cells. **e** The number of colonies formed by cultured liver ECs. Data show mean ± SEM; **p < 0.01 (n > 3). **f** The number of colonies formed by cultured E-SP cells. **g** EC colonies from E-SP cells derived from different tissues. Dashed boxed area is more highly magnified. Scale bars represent 500 μm in **a**, **d**, (**g**, high powered view); 1 mm in (**g**, low powered view)

Because previous research showed that E-SP cells possess stem/progenitor properties [13], we hypothesized that E-SP cells from adipose tissue, skin, and placenta might have highly proliferative ability. We cultured the sorted E-SP cells from different tissues on OP9 stromal cells as feeder cells. After 10 days, we found a “cord-like” network forming EC colonies generated by E-SP cells, but not E-MP cells, both of which were from adipose tissue and skin (Fig. 2a, b). However, we could not detect any endothelial colonies when we cultured placenta-derived E-SP cells. Therefore, these data suggest that adipose tissue and skin are available sources for vascular regeneration.

Furthermore, we also sorted liver E-SP cells, known to have high proliferative and colony-forming ability [13] (Fig. 2c). To compare cells from different tissues, we cultured liver E-SP cells (Fig. 2d), calculated the number of colonies (Fig. 2e), and compared these with adipose tissue- and skin-derived E-SP cells (Fig. 2f). We found that the percentages of SP cells in the adipose tissue and skin are higher than in the liver (Figs. 1b and 2d). However, in adipose tissue- and skin-derived E-SP cells, the proportion of cells which can establish colonies is lower than in liver E-SP cells. In addition, the size of the colonies from the adipose tissue or skin E-SP cells is smaller than from the liver (Fig. 2g).

### Angiogenic ability of E-SP cells and their contribution to neovascularization

**Fig. 3:**
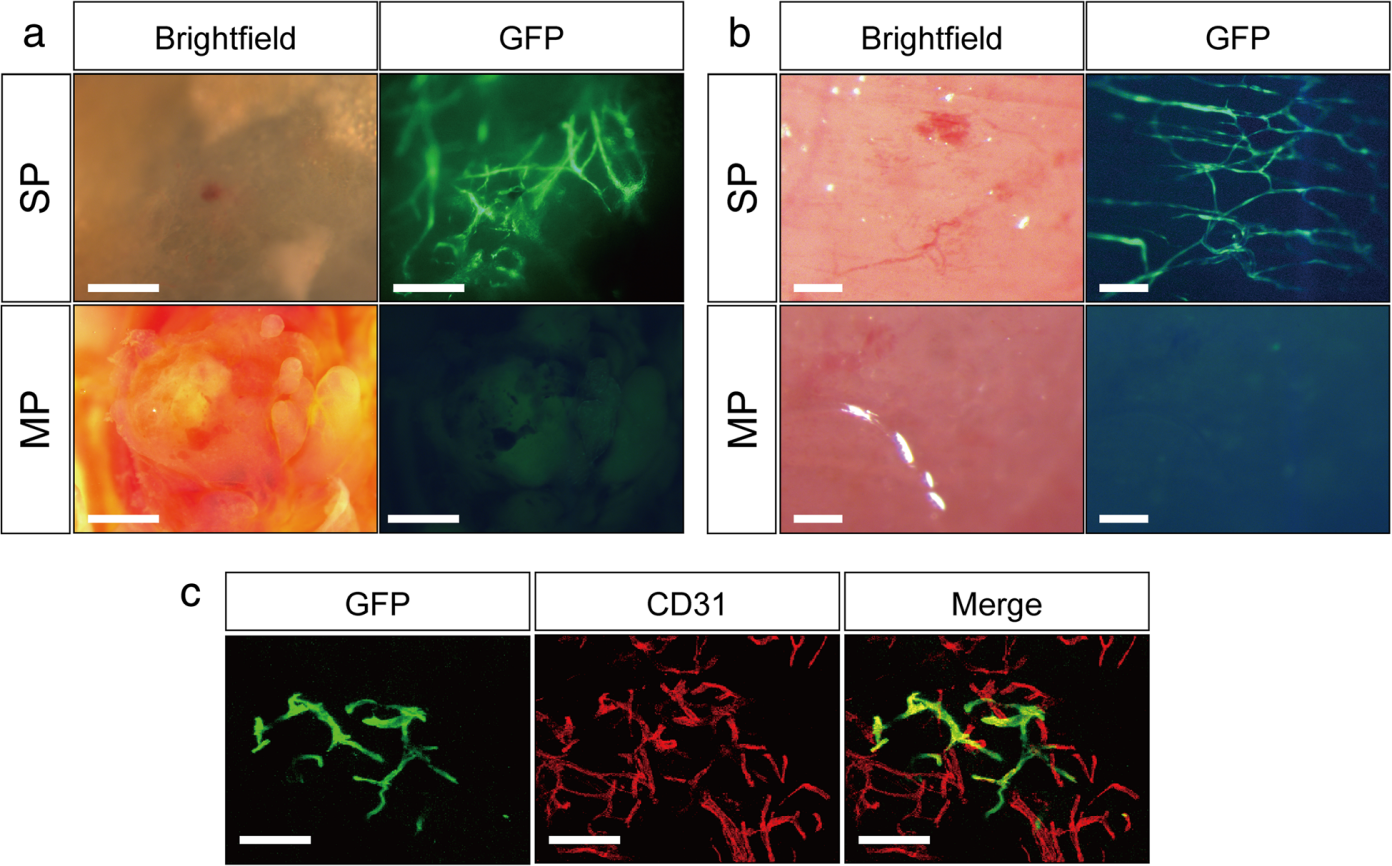
Adipose-derived E-SP cells contribute to regeneration of vasculature in vivo. **a** E-SP and E-MP cells from the adipose tissue of EGFP mice were transplanted into wild-type mice with Matrigel; bar = 500 μm. **b** Fluorescent stereomicroscopic image of hind limb muscle observed 2 weeks after transplantation with E-SP or E-MP cells; bar = 100 μm. **c** Confocal microscopic image of a section from hind limb muscle transplanted with E-SP cells stained with GFP (green) and CD31 (red). The muscle was dissected 2 weeks after transplantation; bar = 100 μm

Next, we observed whether E-SP cells from adipose tissue could contribute to the neovascular formation in an in vivo angiogenesis model. First, we injected E-SP cells mixed with Matrigel in mice subcutaneously and found that E-SP cells formed tube-like structures in Matrigel but E-MP cells did not (Fig. 3a).

Subsequently, we evaluated their contribution to vascular regeneration in a tissue ischemia model. We transplanted E-SP or E-MP cells derived from EGFP mice into ischemic limbs and observed hind limbs of transplanted mice 14 days after transplantation. As shown in Fig. 3b, we confirmed that E-SP cells contributed to the neovascular formation on the hind limb muscle surface but E-MP cells did not. The regenerated vessels were positive for the EC marker CD31 (Fig. 3c). Taken together, our data show that E-SP cells from the adipose tissue can contribute to neovascular regeneration in vivo.

### The origin of E-SP cells is not the BM

**Fig. 4:**
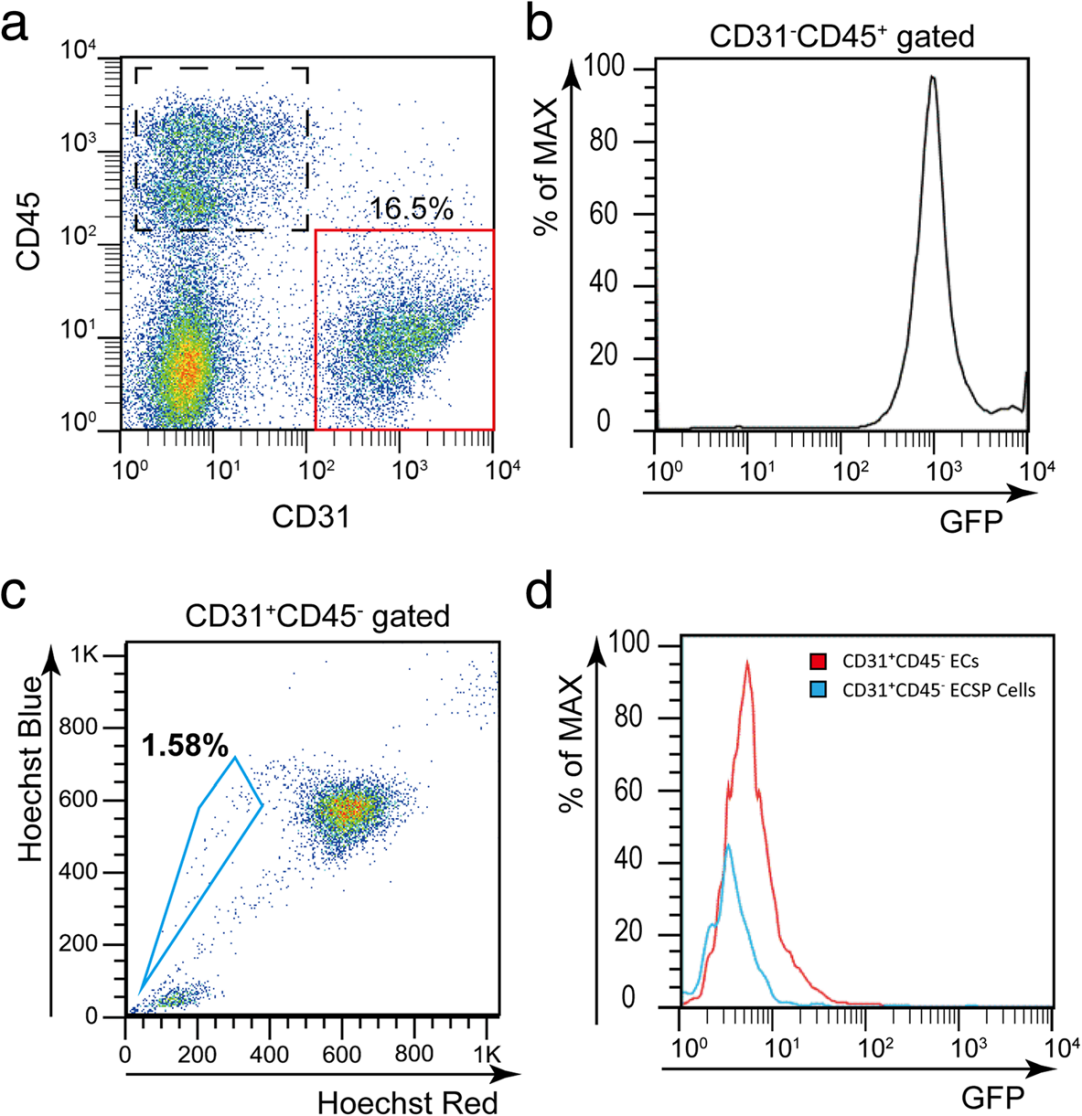
E-SP cells originate from a tissue other than the BM. **a** FACS analysis of cells from the adipose tissue of EGFP-BM transplanted mice. The dashed boxed area indicates CD45^+^CD31^−^ hematopoietic cells, and the red box indicates CD31^+^CD45^−^ ECs. **b** Histogram showing EGFP intensity in hematopoietic cells gated in **a**. **c** Hoechst analysis of ECs gated in **a**. The blue box indicates E-SP cells. **d** Intensity of EGFP in ECs (red line) gated in **a** and E-SP cells (blue line) gated in **c**

We next investigated the origin of E-SP cells. In previous research, hind limb-derived E-SP cells did not originate from the BM [13]. In order to confirm the origin of E-SP cells in the adipose tissue, we conducted BM transplantation: we injected BM cells from EGFP mice into lethally irradiated wild-type mice and observed E-SP cells in the adipose tissue by FACS analysis 3 months after transplantation. When CD31^−^CD45^+^ hematopoietic cells were observed in the adipose tissue, almost all cells were EGFP^+^, suggesting that BM cells were replaced by EGFP^+^ cells (Fig. 4a, b). However, there were no EGFP^+^ cells in CD31^+^CD45^−^ ECs derived from the adipose tissue, and endogenous E-SP cells derived from the adipose tissue were negative for EGFP (Fig. 4a, c, d). Therefore, adipose-derived E-SP cells do not originate from the BM.

### CD157 is highly expressed by E-SP cells

**Fig. 5:**
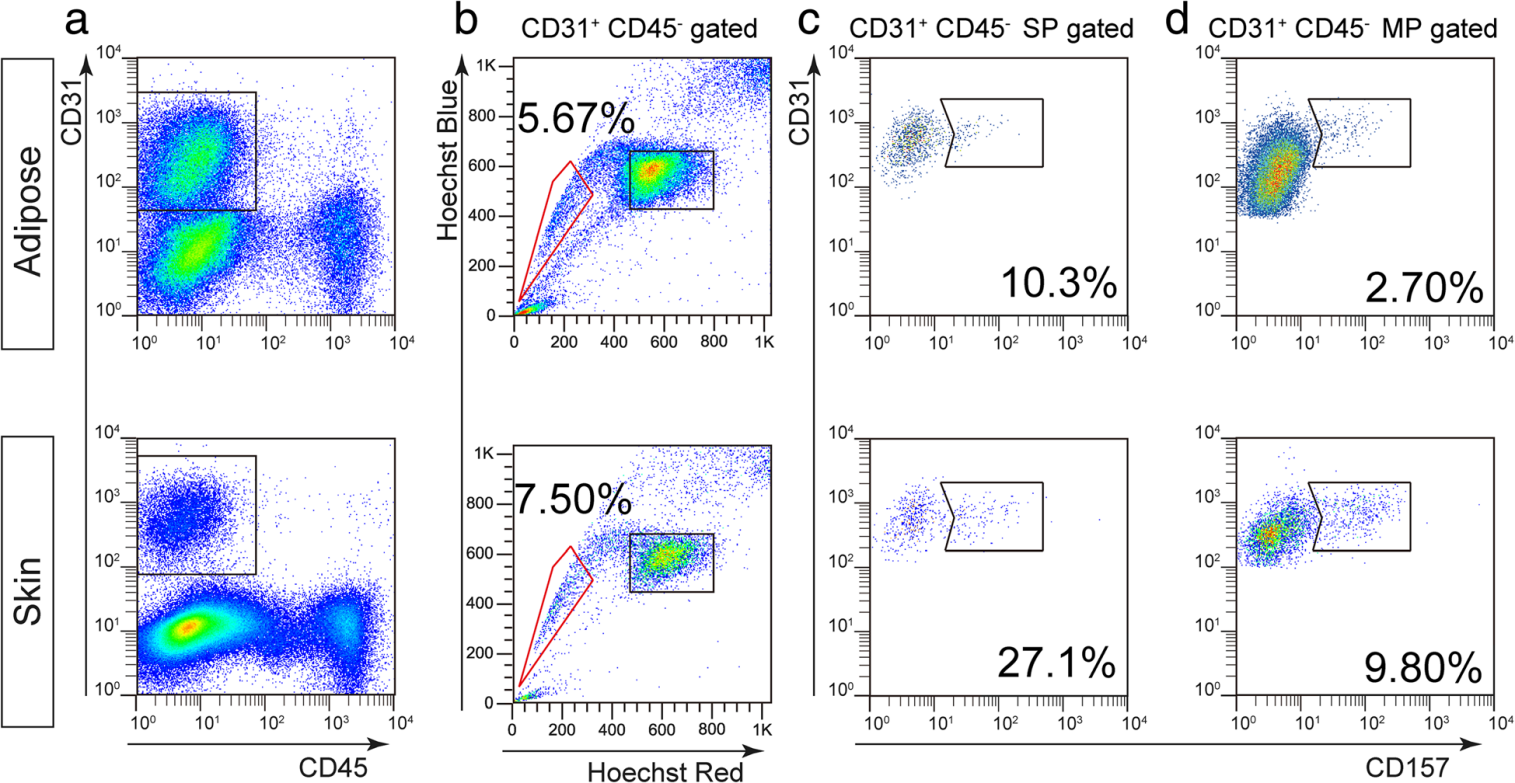
Enrichment of CD157^+^ endothelial stem cells in SP fractions. **a** FACS analysis of ECs from adipose and skin tissues. The boxes indicate CD31^+^CD45^−^ ECs. **b** Hoechst analysis of ECs gated in **a**. The gate surrounded by the red line indicates SP cells and the box indicates MP cells. **c** Analysis of CD157^+^ cells in E-SP cells gated in **b**. **d** Analysis of CD157^+^ cells in E-MP cells gated in **b**

Finally, we examined the relationship between E-SP cells and CD157^+^ vascular endothelial stem cells (VESCs). Previously, we reported that CD157 is highly expressed in E-SP cells and is a marker of VESCs [16]. To assess whether the adipose- or skin-derived E-SP cells express CD157, we performed FACS analysis (Fig. 5a). We found that the percentage of CD157^+^ cells in E-SP populations derived from adipose tissue and skin was 10.3 ± 1.70% and 27.1 ± 2.84%, respectively (Fig. 5b, c). In contrast, the percentage of CD157^+^ E-MP cells derived from these two tissues was 2.70 ± 0.21% and 9.80 ± 1.42%, respectively (Fig. 5b, d). These data indicate that a larger number of adipose tissue and skin E-SP cells are positive for CD157 relative to E-MP cells.

## Discussion

In this report, we show the existence of E-SP cells in adipose tissue, skin, and placenta, good cell sources for tissue regeneration therapy because of their easy accessibility. Although E-SP cells in the adipose tissue and skin contain ECs with highly proliferative ability, those in the placenta lost this ability.

One possible reason of this difference is that the placenta is not permanently required and is excreted after delivery of the baby. Therefore, there is no need to maintain a stem cell population of ECs in the placenta, and endothelial progenitors are only required to generate transient tissue generation. However, we could not completely deny the existence of an endothelial stem cell population in the placenta, and further analyses are required to demonstrate our hypothesis.

Thus far, BM cells have been considered the sources for vascular regeneration [11, 12]. Our study shows that E-SP cells do not originate from the BM. Since the origin of E-SP cells has not been elucidated yet, further analysis on the development of E-SP cells during embryogenesis may clarify their origin.

Comparing the data of a previous report [16] with the present study, although more adipose- or skin-derived E-SP cells expressed CD157 relative to E-MP cells, there remained fewer CD157-positive adipose and skin E-SP cells than present in liver E-SP cells. One possible reason for this difference may be that SP analysis depends on the drug resistance characteristics of the cells analyzed. Thus, the percentage of E-SP cells is different for tissues and organs: for example, in the brain, almost all ECs can extrude Hoechst dye [13, 14]. 54 Therefore, we can enrich endothelial stem cells but cannot purify them completely merely using SP analysis because of functional limitations. Moreover, why fewer colonies are formed by adipose- or skin-derived E-SP cells than by liver-derived E-SP cells is also explicable in this manner. However, further analysis is required to prove that this hypothesis is correct.

Here, we show that E-SP cells from adipose tissue can contribute to the neovascular formation in an in vivo hind limb ischemia model. We previously reported the contribution of E-SP cells from hind limb muscle to neovascular structure in vivo and recovery from ischemia in the hind limb ischemia model [13]. As E-SP cells from adipose tissue have a different origin than E-SP cells from hind limb muscle, E-SP cells from other organs, such as the skin, may also induce neovascularization by participating in angiogenesis in ischemic diseases. Adipose tissue is suggested to be the tissue with easier accessibility and lower invasiveness compared with muscle, retina, and liver, where we previously reported the existence of E-SP cells. Since the adipose tissues are frequently ablated by cosmetic surgery, it can be easily reutilized for clinical use and be a good source of endothelial stem cell population for regenerative therapy.

## Conclusion

Endothelial stem cells exist in the adipose tissue and skin, and the ones in the adipose tissue are available for in vivo neovascular regeneration.

## Abbreviations

BM: Bone marrow
EC: Endothelial cell
E-MP: Endothelial-MP
E-SP: Endothelial-SP
MP: Main population
SP: Side population

## References

[1] Ramasamy SK, Kusumbe AP, Adams RH (2015). Regulation of tissue morphogenesis by endothelial cell-derived signals. Trends Cell Biol.

[2] Hanahan D, Folkman J (1996). Patterns and emerging mechanisms of the angiogenic switch during tumorigenesis. Cell.

[3] Risau W (1997). Mechanisms of angiogenesis. Nature.

[4] Carmeliet P, Jain RK (2011). Molecular mechanisms and clinical applications of angiogenesis. Nature.

[5] Potente M, Gerhardt H, Carmeliet P (2011). Basic and therapeutic aspects of angiogenesis. Cell.

[6] Baumgartner I (1998). Constitutive expression of phVEGF165 after intramuscular gene transfer promotes collateral vessel development in patients with critical limb ischemia. Circulation.

[7] Makino H (2012). Long-term follow-up evaluation of results from clinical trial using hepatocyte growth factor gene to treat severe peripheral arterial disease. Arterioscler Thromb Vasc Biol.

[8] Beenken A, Mohammadi M (2009). The FGF family: biology, pathophysiology and therapy. Nat Rev Drug Discov.

[9] Karantalis V, Hare JM (2015). Use of mesenchymal stem cells for therapy of cardiac disease. Circ Res.

[10] Heldman AW (2014). Transendocardial mesenchymal stem cells and mononuclear bone marrow cells for ischemic cardiomyopathy: the TAC-HFT randomized trial. JAMA.

[11] Asahara T (1997). Isolation of putative progenitor endothelial cells for angiogenesis. Science.

[12] Shantsila E, Watson T, Lip GY (2007). Endothelial progenitor cells in cardiovascular disorders. J Am Coll Cardiol.

[13] Naito H (2012). Identification and characterization of a resident vascular stem/progenitor cell population in preexisting blood vessels. EMBO J.

[14] Wakabayashi T (2013). Identification of vascular endothelial side population cells in the choroidal vessels and their potential role in age-related macular degeneration. Invest Ophthalmol Vis Sci.

[15] Naito H (2016). Endothelial side population cells contribute to tumor angiogenesis and antiangiogenic drug resistance. Cancer Res.

[16] Wakabayashi T (2018). CD157 marks tissue-resident endothelial stem cells with homeostatic and regenerative properties. Cell Stem Cell.

[17] Goodell MA (1996). Isolation and functional properties of murine hematopoietic stem cells that are replicating in vivo. J Exp Med.

[18] Takakura N (2000). A role for hematopoietic stem cells in promoting angiogenesis. Cell.

[19] Takara K (2017). Lysophosphatidic acid receptor 4 activation augments drug delivery in tumors by tightening endothelial cell-cell contact. Cell Rep.

[20] Kidoya H, Naito H, Takakura N (2010). Apelin induces enlarged and nonleaky blood vessels for functional recovery from ischemia. Blood.

